# Supplementary material for: Exploring neural oscillations during speech perception via surrogate gradient spiking neural networks
Source: Front Neurosci. 2024 Sep 25;18:1449181. doi: 10.3389/fnins.2024.1449181 (PMC11461475; doi:10.3389/fnins.2024.1449181)
Supplement: Supplementary file 1 [file Data_Sheet_1.pdf]

Supplementary Material

1 APPENDIX

1.1 Forward-Euler first-order exponential integrator method

Consider the following differential equation,

$$\tau\,\dot{y} = -y + \mathcal{N}(y) + \tau\,\gamma\,s(t)\,, \tag{S1}$$

where $\mathcal{N}(y)$ is a nonlinear function and $s(t)$ is a spike train defined as

$$s(t) = \sum_f \delta(t - t^f)\,. \tag{S2}$$

Multiplying both sides by $\frac{1}{\tau}\exp(\frac{t}{\tau})$ and then integrating over $[t_n, t_{n+1}]$, where $t_n = n\,\Delta t$, yields

$$\int_{t_n}^{t_{n+1}} \Big(\dot{y}\exp\frac{t}{\tau} + y\,\frac{1}{\tau}\,\exp\frac{t}{\tau}\Big)dt = \frac{1}{\tau}\int_{t_n}^{t_{n+1}} \mathcal{N}(y)\exp\frac{t}{\tau}\,dt + \gamma\sum_f\int_{t_n}^{t_{n+1}} \exp\frac{t}{\tau}\,\delta(t - t^f)\,. \tag{S3}$$

The left hand side has an exact solution

$$y(t)\,\exp\frac{t}{\tau}\bigg|_{t=t_n}^{t_{n+1}} = \Big(y_{n+1}\exp\frac{\Delta t}{\tau} - y_n\Big)\exp\frac{t_n}{\tau}\,. \tag{S4}$$

For the first term of the right hand side, the nonlinearity $\mathcal{N}(y)$ can be approximated as constant over $[t_n, t_{n+1}]$ for sufficiently small Δt, so that $\mathcal{N}(y) \approx \mathcal{N}(y_n)$ and we can solve it as

$$\frac{1}{\tau}\int_{t_n}^{t_{n+1}} \mathcal{N}(y)\exp\frac{t}{\tau}\,dt \approx \mathcal{N}(y_n)\,\exp\frac{t}{\tau}\bigg|_{t=t_n}^{t_{n+1}} = \mathcal{N}(y_n)\exp\frac{t_n}{\tau}\Big(\exp\frac{\Delta t}{\tau} - 1\Big)\,. \tag{S5}$$

Finally for the last term, the width Δt of the interval $[t_n, t_{n+1}]$ can be set sufficiently small to include at most a single spike. The exact firing time $t^f \in [t_n, t_{n+1}]$ can then be discretised as $t^f = t_n$ so that $s_n = \sum_f \delta(t_n - t^f)$ and

$$\gamma\sum_f \exp\frac{t^f}{\tau}\bigg|_{t^f\in[t_n,t_{n+1}]} = \gamma\exp\frac{t^n}{\tau}\,s_n \tag{S6}$$

Putting everything together, we get the following update equation for y in discrete time,

$$y_{n+1} = \exp\frac{-\Delta t}{\tau}\Big(y_n + \gamma\,s_n\Big) + \Big(1 - \exp\frac{-\Delta t}{\tau}\Big)\,\mathcal{N}(y_n)\,. \tag{S7}$$

1.2 Eigenvalues of AdLIF free equations

The free equations of the AdLIF neuron model are obtained by considering Eqs. (3) and (4) in the special case where there is no input, $I(t) = 0$, and no emitted spikes, $s(t) = 0$. They can be rewritten in matrix

form as,

$$\frac{d}{dt}\begin{bmatrix}u\\w\end{bmatrix}=\begin{bmatrix}-1/\tau_u & -1/\tau_u\\ a/\tau_w & -1/\tau_w\end{bmatrix}\begin{bmatrix}u\\w\end{bmatrix}=A\begin{bmatrix}u\\w\end{bmatrix}. \tag{S8}$$

The eigenvalues can be found by setting the determinant of $A-\lambda\mathbb{I}$ to zero,

$$\begin{vmatrix}-1/\tau_u-\lambda & -1/\tau_u\\ a/\tau_w & -1/\tau_w-\lambda\end{vmatrix}=0\,, \tag{S9}$$

yielding the characteristic polynomial,

$$\lambda^2+\lambda\left(\frac{1}{\tau_u}+\frac{1}{\tau_w}\right)+\frac{1+a}{\tau_u\tau_w}=0\,, \tag{S10}$$

whose roots correspond to the two eigenvalues of the system,

$$\lambda_{1,2}=-\frac{1}{2}\left(\frac{1}{\tau_u}+\frac{1}{\tau_w}\right)\pm\frac{1}{2}\sqrt{\left(\frac{1}{\tau_u}+\frac{1}{\tau_w}\right)^2-\frac{4(1+a)}{\tau_u\tau_w}}\,. \tag{S11}$$

In order to prevent the occurrence of exponentially growing solutions and ensure stability, both eigenvalues need to have a strictly negative real part, which can be realised by imposing a lower bound $a>-1$ on the coupling strength. Moreover, allowing eigenvalues to have a nonzero imaginary part introduces the potential for oscillatory modes that may amplify perturbations. This could cause some challenges in terms of numerical stability, convergence and interpretability, especially in the context of deep neural networks trained with gradient descent. We therefore impose an additional upper bound on the values of a leading to the overall stability condition,

$$-1<a\leq\frac{\left(\tau_w-\tau_u\right)^2}{4\tau_u\tau_w}\,. \tag{S12}$$

1.3 Kernel formulation of a spiking neuron

Using the SRM formulation, the membrane potential $u(t)$ is described as,

$$u(t)=\int_0^\infty \kappa(s)\,I(t-s)ds+\int_0^\infty \eta(s)\,S(t-s)ds\,, \tag{S13}$$

where the two kernels $\kappa(s)$ and $\eta(s)$ describe the response to an input pulse and the response to an afterspike reset pulse respectively. It can be shown that the differential equations of a linear AdLIF neuron has an equivalent kernel formulation with,

$$\kappa(s)=\left(\beta_1\,e^{\lambda_1 s}+\beta_2\,e^{\lambda_2 s}\right)\Theta(s) \tag{S14a}$$

$$\eta(s)=\left(\gamma_1\,e^{\lambda_1 s}+\gamma_2\,e^{\lambda_2 s}\right)\Theta(s)\,, \tag{S14b}$$

where λ_1, λ_2 are the eigenvalues of the system given in Supplementary Eq. (S11) and $\Theta(s)$ is the Heaviside step function. The coefficients β_1, β_2 of the input kernel are such that the membrane potential increases by

$\Delta u = 1$, without any effect on the recovery current, i.e., $\Delta w = 0$,

$$\beta_1 = \frac{\tau_u \lambda_2 + 1}{\tau_u(\lambda_2 - \lambda_1)} \quad \text{and} \quad \beta_2 = 1 - \beta_1 \, . \tag{S15}$$

The coefficients γ_1, γ_2 on the afterspike reset kernel are such that the membrane potential decreases by $\Delta u = \vartheta - u_r$ and the recovery current jumps by an amount $\Delta w = b$,

$$\gamma_1 = \frac{b - (\vartheta - u_r)(\tau_u \lambda_2 + 1)}{\tau_u(\lambda_2 - \lambda_1)} \quad \text{and} \quad \gamma_2 = -(\vartheta - u_r) - \gamma_1 \, . \tag{S16}$$

2 SUPPLEMENTARY TABLES

Table S1. Hyperparameter tuning for the simulation time step on the TIMIT dataset. PERs are reported after 50 epochs using 16 CNN channels, 3 layers of 512 neurons each, 50% of AdLIF neurons, 100% feedforward and 50% recurrent connectivity.

Time step [ms]	Epoch duration [min]	Test PER [%]	Validation PER [%]
5	21	20.5	18.2
2	53	20.4	18.7
1	156	20.6	18.2

Table S2. Hyperparameter tuning for the number of CNN channels on the TIMIT dataset. PERs are reported after 50 epochs using a 5 ms time step, 3 layers, 512 neurons per layer, 50% of AdLIF neurons, 100% feedforward and 50% recurrent connectivity. Bold values indicate the lowest achieved PERs.

CNN channels	Nerve fibers	Parameters in complete encoder	Test PER [%]	Validation PER [%]
8	592	1.8M	20.9	18.9
16	1,184	2.1M	20.5	18.2
32	2,368	2.7M	20.2	18.4
64	4,736	3.9M	**19.8**	**18.0**
128	9,472	6.4M	21.3	18.9

Table S3. Hyperparameter tuning for the feedforward connectivity on the TIMIT dataset. PERs are reported after 50 epochs using a 5 ms time step, 16 CNN channels, 3 layers of 512 neurons each, 50% of AdLIF neurons and 50% recurrent connectivity. Bold values indicate the lowest achieved PERs.

Feedforward connectivity	Number of parameters	Test PER [%]	Validation PER [%]
0.2	629k / 1.2M	22.0	19.6
0.5	968k / 1.5M	21.6	19.7
0.8	1.3M / 1.8M	20.7	18.8
1.0	1.5M / 2.1M	**20.5**	**18.2**

REFERENCES

Devi, N., Sreeraj, K., Anuroopa, S., Ankitha, S., and Namitha, V. (2022). Q10 and tip frequencies in individuals with normal-hearing sensitivity and sensorineural hearing loss. *Indian Journal of Otology* 28, 126–129. doi:10.4103/indianjotol.indianjotol_5_22

Table S4. Hyperparameter tuning for trainable delays on the TIMIT dataset. PERs are reported after 50 epochs using a 5 ms time step, 16 CNN channels, 3 layers of 512 neurons. Bold values indicate the lowest achieved PERs.

Model type	Conv Groups	Number of parameters	Test PER [%]	Validation PER [%]
No recurrence no SFA	1	2.3M / 2.8M	26.2	24.6
Recurrence only	1	2.7M / 3.2M	21.6	19.5
SFA only	1	2.3M / 2.8M	23.3	20.7
Recurrence and SFA	1	2.7M / 3.2M	**20.8**	18.6
Recurrence and SFA	4	968k / 1.5M	21.0	**18.4**

Table S5. Results with moving threshold SFA on the TIMIT dataset. PERs are reported after 50 epochs using a 2 ms time step, 16 CNN channels, 3 layers of 512 neurons.

Model type	Number of parameters	Test PER [%]	Validation PER [%]
SFA only	1.1 / 1.7M	26.3	23.9
Recurrence and SFA	1.5 / 2.1M	22.1	19.2

Table S6. Sensitivity to frequency of auditory nerve fibers. The second column gives the sensitivity ranges measured in Devi et al. (2022) that we compute from their reported mean Q10 values in the normal hearing group. The third column gives the Mel scale centers using 80 filters that surround the corresponding sensitivity ranges.

Characteristic Frequency [Hz]	Sensitivity range [Hz]	Nearby Mel bin centers [Hz]	Overlapping Number of bins
500	416-583	416, 452, 488, 525, 564, 604	5-6
1000	881-1119	872, 921, 973, 1026, 1080, 1136	5-6
2000	1770-2230	1729, 1806, 1886, 1967, 2052, 2139, 2228	6-7
4000	3566-4434	3553, 3688, 3827, 3970, 4117, 4270, 4427	7
6000	5501-6499	5479, 5674, 5875, 6083, 6297, 6519	5-6
